# Two new corticioid species in Amylocorticiales and Atheliales (Agaricomycetes, Basidiomycota) from Southwestern China

**DOI:** 10.3897/mycokeys.134.200816

**Published:** 2026-06-18

**Authors:** Yu-Jin Cui, Chao-Ge Wang, Yu-Cheng Dai, Ying-Da Wu

**Affiliations:** 1 School of Ecology and Nature Conservation, Beijing Forestry University, Beijing 100083, China School of Ecology and Nature Conservation, Beijing Forestry University Beijing China; 2 School of Life Sciences, Henan University, Zhengzhou, 450046, China School of Life Sciences, Henan University Zhengzhou China; 3 Key Laboratory of Ministry of Emergency Management for Forest and Grassland Fire Risk Prevention, China Fire and Rescue Institute, Beijing 102202, China Key Laboratory of Ministry of Emergency Management for Forest and Grassland Fire Risk Prevention, China Fire and Rescue Institute Beijing China

**Keywords:** corticioid fungi, *
Leptosporomyces
*, new taxa, *
Pseudoathelia
*, wood-decaying fungi

## Abstract

Amylocorticiales and Atheliales (Basidiomycota) comprise corticioid, athelioid, and poroid fungi, characterized by smooth to pellicular hymenophores, a monomitic hyphal system, and smooth basidiospores. In this study, two new species from southwestern China, namely *Leptosporomyces caeruleogriseum* and *Pseudoathelia fabri*, are described and illustrated based on morphology characteristics and phylogenetic analyses of ITS and nLSU sequences. *Leptosporomyces caeruleogriseum* is characterized by coriaceous basidiomata with a bluish gray hymenophore surface when fresh, becoming soft membranous and cream to curry yellow upon drying, as well as by encrusted generative hyphae, and basidiospores are oblong ellipsoid, measuring 3–4 × 1.5–2 µm, and the species is currently known from Guizhou and Xizang, China. *Pseudoathelia fabri* is distinguished by a merulioid to tuberculate hymenophore with a white surface when fresh, a fimbriate sterile margin, and oblong ellipsoid basidiospores measuring 4.1–5.5 × 1.8–2.4 µm. To date, it is currently known only from Xizang, China. Phylogenetically, the new species *Leptosporomyces caeruleogriseum* was closely related to *L. galzinii*, the type species of *Leptosporomyces*, and forming independent clade in Atheliales, and *Pseudoathelia fabri* formed an independent lineage within the *Pseudoathelia* clade in Amylocorticiales.

## Introduction

Amylocorticiales K.H. Larss., Manfr. Binder & Hibbett and Atheliales Jülich, Biblioth are relatively small fungal orders within Agaricomycetes Doweld (Basidiomycota), mainly comprising corticioid, athelioid, and poroid fungi with resupinate basidiomata, a monomitic hyphal system, and smooth basidiospores (Dai et al. 2004; Sulistyo et al. 2021; Liu et al. 2026; Yuan et al. 2026). Although species diversity in both orders is relatively limited compared with that of other Agaricomycetes groups, their taxonomy and phylogenetic relationships have attracted increasing attention in recent years owing to the rapid development of molecular systematics.

Amylocorticiales was established by Binder et al. (2010) within the subclass Agaricomycetidae Parmasto together with Agaricales Underw, Atheliales, Boletales E.-J. Gilbert, and Jaapiales Manfr. Binder, K.H. Larss. & Hibbett. Then, the order Jaapiales was removed from Agaricomycetidae based on phylogenomic analyses (He et al. 2024). In recent years, phylogenetic studies based on multigene datasets have substantially improved the taxonomy of Amylocorticiales, leading to the reassignment of some polyphyletic genera into newly erected monophyletic groups based on phylogenetic analyses. For example, Palla et al. (2024) revised the systematic position of *Irpicodon* Pouzar, *Plicaturopsis* D.A. Reid, and *Plicatura* Peck, in which the type species of these genera formed a clade and shared similar morphological characteristics as pendant fan-shaped effused-reflexed basidiomata. Consequently, *Irpicodon* and *Plicaturopsis* were synonymized with *Plicatura*. In addition, *Pseudoathelia* Yu Q. Liu & S.H. He, typified by *P. septentrionalis* (J. Erikss.) Yu Q. Liu & S.H. He (basionym: *Athelia septentrionalis* J. Erikss.), was segregated from *Leptosporomyces* Jülich as an independent genus because the genus formed a new clade within the Amylocorticiales (Liu et al. 2026).

Amylocorticiales have a single family only; the family is typified by *Amylocorticium* Pouzar and include fourteen genera (He et al. 2019; Yuan et al. 2020; Palla et al. 2024; Liu et al. 2026). The exceptions are *Anomoloma* Niemelä & K.H. Larss. and *Anomoporia* Pouzar, which are characterized by poroid hymenophores (Niemelä et al. 2007; Zhou et al. 2022); all remaining genera in the order are corticioid fungi, including *Agroathelia* Redhead & Mullineux, *Amyloathelia* Hjortstam & Ryvarden, *Amyloceraceomyces* S.H. He, *Amylocorticiellum* Spirin & Zmitr., *Amylocorticium* Pouzar, *Amylophanerochaete* Yu Q. Liu & S.H. He, *Amyloxenasma* (Oberw.) Hjortstam & Ryvarden, *Ceraceomyces* Jülich, *Plicatura* Peck, *Podoserpula* D.A. Reid, *Pseudoathelia* Yu Q. Liu & S.H. He, and *Serpulomyces* (Zmitr.) Zmitr.

Atheliales was established by Jülich (1982) and originally included four families, namely Atheliaceae Jülich, Byssocorticiaceae Jülich, Pilodermataceae Jülich, and Tylosporaceae Jülich. More than 100 species belonging to 20 genera have been reported within the order (He et al. 2024; Hyde et al. 2024; Yuan et al. 2026). Sulistyo et al. (2021) reconstructed the phylogeny of Atheliales based on ten genera and introduced a new family Lobuliciaceae Sulistyo, K.H. Larss. & M. Ryberg, typified by *Lobulicium* K.H. Larss. & Hjortstam. Subsequently, *Atheliella* S.L. Liu & L.W. Zhou was proposed as a new genus based on its pellicular and cracked basidiomata, as well as an independent phylogenetic position within the order (Liu et al. 2024).

Currently, eleven genera are accepted in Atheliales, viz. *Amphinema* P. Karst., *Athelia* Pers., *Atheliella*, *Athelopsis* Oberw. ex Parmasto, *Byssocorticium* Bondartsev & Singer, *Fibulomyces* Jülich, *Leptosporomyces* Jülich, *Lobulicium*, *Piloderma* Jülich, *Tretomyces* K.H. Larss., Kotir. & Saaren., and *Tylospora* Donk (Hyde et al. 2024). Species of Atheliales exhibit diverse life strategies, including saprotrophic, symbiotic, and parasitic lifestyles. Among them, *Amphinema*, *Byssocorticium*, *Piloderma*, and *Tylospora*, are ectomycorrhizal genera (Tedersoo et al. 2010; Palla et al. 2024). In contrast, many numbers of *Athelia*, *Atheliella*, *Athelopsis*, *Fibulomyces*, and *Leptosporomyces* are mainly wood- or lichen-inhabiting saprotrophs (Tedersoo et al. 2014; Sulistyo et al. 2021), whereas some species of *Athelia* are known to parasitize lichens and termites (Yurchenko and Olubkov 2003; Maekawa et al. 2020).

*Athelopsis* and *Leptosporomyces* are typified by *A. glaucina* (Bourdot & Galzin) Oberw. ex Parmasto and *L. galzinii* (Bourdot) Jülich, respectively. *Athelopsis* is characterized by the pellicular and smooth basidiomata, hymenophore with pale yellowish to green tints, a monomitic hyphal system with clamps on generative hyphae, and ellipsoid to cylindrical basidiospores (Eriksson and Ryvarden 1973; Gorjón et al. 2012), and *Leptosporomyces* is characterized by the pellicular and smooth basidiomata, whitish hymenophore or with some green or pinkish tints on the surface, generative hyphae bearing clamps connections, short-cylindrical basidia, and ellipsoid to cylindrical basidiospores (Eriksson and Ryvarden 1976; Jülich 1982; Zhou et al. 2024). Up to now, 17 and 13 species have been accepted in *Athelopsis* and *Leptosporomyces* respectively in Index Fungorum (http://www.indexfungorum.org/). Recent phylogenetic studies have suggested that both genera were polyphyletic (Hodkinson et al. 2014; Sulistyo et al. 2021; Zhou et al. 2024). Several species currently assigned to these genera, including *A. subinconspicua* (Litsch.) Jülich, *L. raunkiaeri* (M.P. Christ.) Jülich, and *L. fuscostratus* (Burt) Hjortstam, formed an independent clade within Atheliales based on the loci of ITS, nLSU. (Hodkinson et al. 2014; Sulistyo et al. 2021; Zhou et al. 2024).

During investigations of corticioid fungi in southwestern China, some new specimens belonging to *Leptosporomyces* and *Pseudoathelia* were discovered in Guizhou and Xizang, China. Their taxonomic positions were confirmed based on morphological characteristics and phylogenetic analyses of the combined ITS+nLSU dataset, and the two new species are described and illustrated herein.

## Materials and methods

### Morphological studies

The studied specimens are deposited in the fungarium of the institute of Microbiology, Beijing Forestry University (BJFC). Morphological descriptions are based on field notes and voucher specimens. Microscopic procedures followed Dai (2010) and Wu et al. (2022). Sections were studied at a magnification of up to 1,000 × using a Nikon Eclipse 80i microscope and phase contrast illumination. Descriptions of microscopic features and measurements were made from slide preparations stained with Cotton Blue and Melzer’s reagent. Basidiospores were measured from sections cut from the hymenial surface. In presenting the variation in basidiospore size, 5% of the measurements from each end of the range were excluded, and the excluded values are given in parentheses. In the description: KOH = 5% potassium hydroxide, IKI = Melzer’s reagent, IKI– = neither amyloid nor dextrinoid, CB = Cotton Blue, CB– = acyanophilous in Cotton Blue, CB+ = cyanophilous in Cotton Blue, L = arithmetic average of spore length, W = arithmetic average of spore width, Q = L/W ratios, and n (a/b) = number of basidiospores (a) measured from the given number of specimens (b). Colour terms follow Petersen (1996).

### DNA extraction, amplification, and sequencing

A CTAB rapid plant genome extraction kit-DN14 (Aidlab Biotechnologies Co., Ltd, Beijing) was used to obtain DNA from dried specimens, followed by the polymerase chain reaction (PCR) according to the manufacturer’s instructions with some modifications (Sun et al. 2020; Qin et al. 2025). The nuclear ribosomal internal transcribed spacer regions (ITS) and the large subunit nuclear.

RNA gene (nLSU) were amplified using the primer pairs ITS5/ITS4 and LR0R/LR7, respectively (White et al. 1990; Hopple and Vilgalys 1999). The PCR procedure for ITS was as follows: initial denaturation at 95 °C for 3 min, followed by 34 cycles at 94 °C for 40 s, annealing at 54 °C for 45 s and extension at 72 °C for 1 min, and a final extension of 72 °C for 10 min. The PCR procedure for nLSU was as follows: initial denaturation at 94 °C for 1 min, followed by 34 cycles of denaturation at 94 °C for 30 s, annealing at 50 °C for 1 min and extension at 72 °C for 1.5 min, and a final extension at 72 °C for 10 min. The PCR products were purified and sequenced at the Beijing Genomics Institute (BGI), China, with the same primers as used in PCR. Newly generated sequences were deposited in GenBank (Sayers et al. 2023). All sequences analysed in this study are listed in Suppl. material 1.

### Sequence alignment

Newly generated sequences were checked for ambiguous bases and assembled in BioEdit v.7.7.1.0 (Hall 1999). The newly generated sequences, together with additional sequences retrieved from GenBank, were aligned separately for the ITS and nLSU datasets using MAFFT v.7 with the E-INS-i algorithm (Katoh and Standley 2013). The resulting alignments were manually inspected and adjusted in BioEdit. Ambiguously aligned regions were excluded prior to phylogenetic analyses. The aligned datasets were concatenated and converted into different formats in Mesquite v.3.2 (Maddison and Maddison 2017).

### Phylogenetic analyses

In this study, a combined ITS+nLSU dataset was reconstructed to determine the phylogenetic positions of the new species. The sequence alignments and the retrieved topologies were deposited in TreeBase (http://www.treebase.org), under accession ID: 32654 (Reviewer access URL: http://purl.org/phylo/treebase/phylows/study/TB2:S32532?x-access-code=74c447e7541163ef3f4cefaf104f2b6&format=html). *Lactarius deceptivus* Peck and *Russula emeticicolor* Jul. Schäff. retrieved from GenBank were selected as outgroups taxa following Sulistyo et al. (2021). Phylogenetic analyses followed the methods of Han et al. (2016) and Zhu et al. (2019). Maximum Likelihood (ML) and Bayesian Inference (BI) analyses were conducted based on the combined ITS + nLSU dataset. The combined dataset was partitioned into four subsets (ITS1, 5.8S, ITS2, nLSU). The best-fit substitution model for each partition was selected under Akaike information criterion (AIC) using PartitionFinder within PhyloSuite v1.2.3 (Lanfear et al. 2017; Zhang et al. 2020). BI was performed using a partitioned mixed-model approach with separate model settings for ITS1, 5.8S, ITS2, and nLSU.

**Figure 1.**
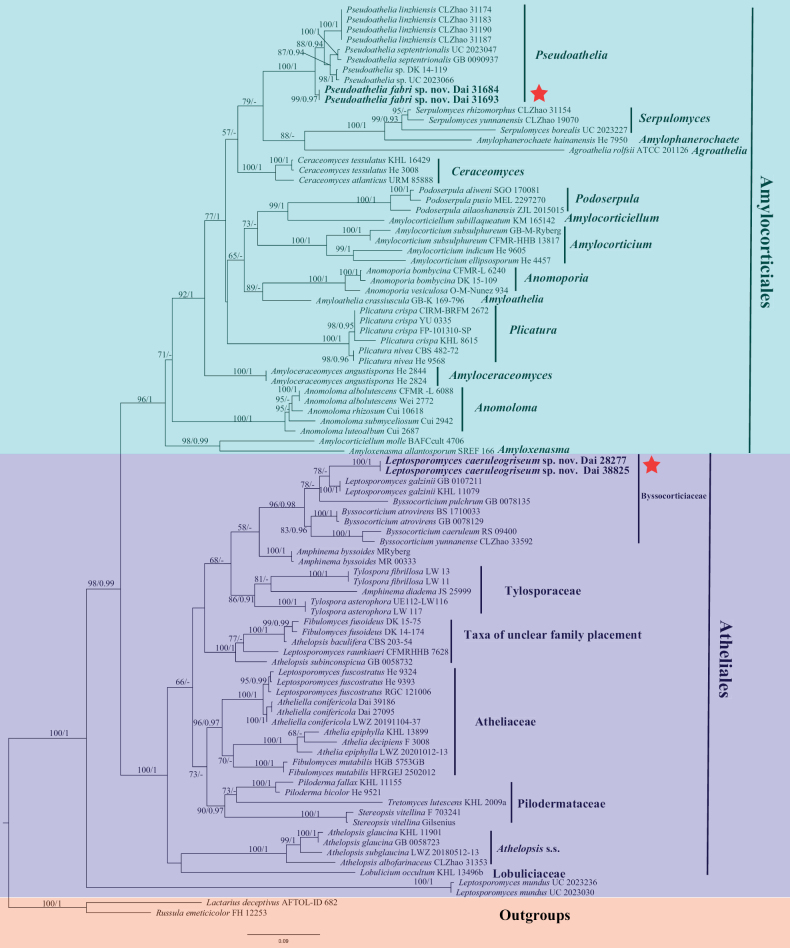
ML tree of Amylocorticiales and Atheliales based on the combined ITS+nLSU dataset. BS values ≥ 50% and BPPs values ≥ 0.90 are shown. Newly described species are marked with red stars. The scale bar represents the estimated number of substitutions per site.

Phylogenetic analyses were conducted using ML in RAxML-HPC v.8.2.12 through the CIPRES Science Gateway (Miller et al. 2010). Branch support for ML analysis was determined by 1,000 bootstrap replicates. BI and Bayesian posterior probabilities (BPPs) were computed with MrBayes 3.2.6 (Ronquist et al. 2012). Four Markov chains were run for 3 million generations until the average standard deviation of split frequencies fell below 0.01, and trees were sampled every 100 generations. The first 25% of the sampled trees were discarded as burn-in, whereas the remaining trees were used to construct a majority-rule consensus tree and to calculate Bayesian posterior probabilities. All phylogenetic trees were visualized in FigTree v. 1.4.3 (http://tree.bio.ed.ac.uk/software/figtree/). Branches receiving ML bootstrap support values (BS) ≥ 75%, and Bayesian posterior probabilities (BPPs)≥ 0.95 were considered significantly supported. BS ≥ 50% and BPPs ≥ 0.90 shown on the ML topology in Fig. 1.

## Results

### BLAST analysis of ITS sequences for these new species

BLAST searches of the ITS sequences from the holotype specimens against the GenBank database showed that the sequence of *Leptosporomyces caeruleogriseum* (Dai 38825) was the most similar to the sequence of *Leptosporomyces* sp. (OL437007), with 91.93% identity (100% query cover). The sequence of *Pseudoathelia fabri* (Dai 31684) was the most similar to *Leptosporomyces* sp. (OL436794), with 96.21% identity (100% query cover). The nLSU sequence of *P. fabri* (Dai 31684) showed the highest similarity to *Pseudoathelia linzhiensis* (C.L. Zhao & H.M. Zhou) Yu Q. Liu & S.H. He (PP862922; deposited as *Leptosporomyces linzhiensis* in GenBank), with 98.37% sequence identity and 100% query coverage. These relatively low to moderate sequence similarities, together with the phylogenetic and morphological evidence, support the recognition of *L. caeruleogriseum* and *P. fabri* as two independent species.

### Molecular phylogeny

The combined ITS+nLSU dataset comprised sequences from 91 samples representing 60 taxa and contained 2,287 aligned characters, of which 745 (33%) were parsimony informative. Phylogenetic analyses inferred from ML and BI produced similar topologies with only minor differences in statistical support. The substitution model employed for ML was GTRGAMMA, and the combined dataset was partitioned into four subsets (ITS1, 5.8S, ITS2, and nLSU). The best-fit models selected for BI were GTR+I+G for ITS1, K81+G for 5.8S, GTR+I+G for ITS2, and GTR+I+G for nLSU. Because the BI and ML analyses generated nearly identical topologies, only the ML tree is presented in Fig. 1.

The phylogeny tree (Fig. 1) illustrates the taxonomic relationships among the accepted genera in Amylocorticiales and Atheliales. *Athelopsis*, *Fibulomyces*, and *Leptosporomyces* are polyphyletic genera. The new species *Pseudoathelia fabri* formed an independent lineage within the *Pseudoathelia* clade in Amylocorticiales and was distinct from the other two species, *P. linzhiensis* and *P. septentrionalis*. *Leptosporomyces caeruleogriseum* was closely related to *L. galzinii*, the type species of *Leptosporomyces*, and formed an independent clade (100% ML, 1.00 BPP, Fig. 1).

In the present phylogeny, the type species of *Byssocorticium* and *Leptosporomyces* formed a well-supported clade (96% ML, 0.98 BPP, Fig. 1), including five other additional species, viz. *Byssocorticium atrovirens* (Fr.) Bondartsev & Singer, *Byssocorticium caeruleum* Kotir., Saaren. & K.H. Larss, *B. pulchrum* (S. Lundell) M.P. Christ, *B. yunnanense* Qi Yuan & C.L. Zhao, and *L. caeruleogriseum*. However, the polyphyly of *Leptosporomyces* has been demonstrated in previous studies (Larsson 2007; Hodkinson et al. 2014; Sulistyo et al. 2021; Liu et al. 2024). Therefore, *Byssocorticium* and *Leptosporomyces* are retained as separate genera in the present study. Two specimens collected from southwestern China (Dai 28277 and Dai 38825) are described herein as a new species of *Leptosporomyces*.

### Taxonomy

#### Leptosporomyces

Taxon classification

Fungi

AthelialesAtheliaceae

Jülich, Willdenowia, Beih. 7: 192 (1972)

2C6D096B-2634-5F73-B5A2-EDBBEA06942E

##### Notes.

The genus *Leptosporomyces*, typified by *L. galzinii*, was established by Jülich (1972). The species of this genus are generally inconspicuous in micromorphology, with resupinate basidiomata and light-colored smooth hymenophore. Currently, fourteen species under the genus are accepted in Index Fungorum (http://www.indexfungorum.org/).

#### Leptosporomyces caeruleogriseum

Taxon classification

Fungi

AthelialesAtheliaceae

Y.J. Cui, Yuan Yuan, Chao G. Wang & Y.C. Dai sp. nov.

19EDFA4D-1B3D-54C2-9178-74C9BCDD56F2

863719

Figs 2, 3

##### Holotype.

China • Xizang, Linzhi, Bayi Dist., Forest along the Road 318, 30°06.138'N, 95°06.624'E, elevation 2,100 m, on fallen branch of *Pinus yunnanensis*, 7 October 2025, Dai 38825 (BJFC060084).

**Figure 2.**
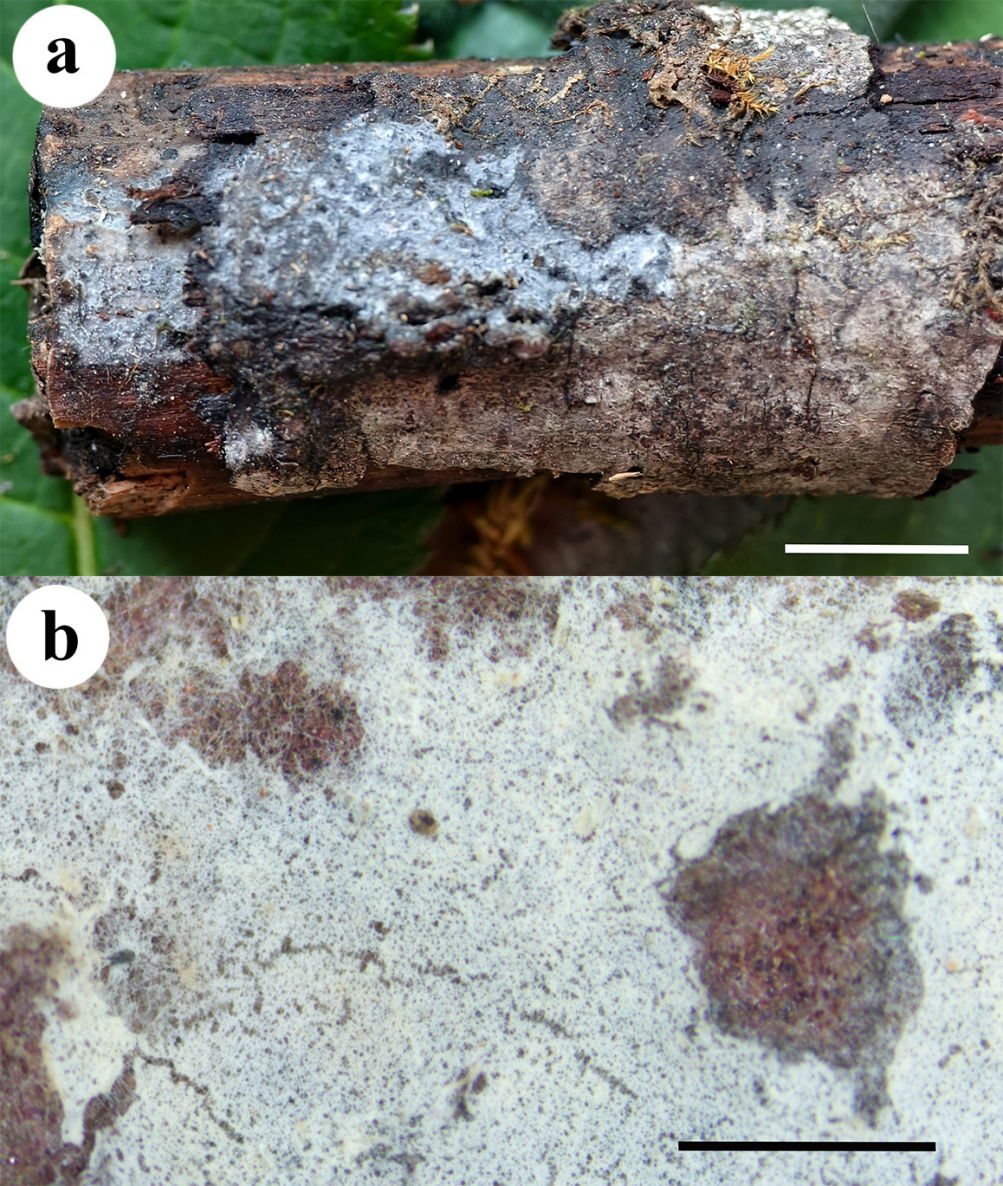
Basidiomata of *Leptosporomyces caeruleogriseum* (holotype, BJFC 060084). Scale bars: 1 cm (**a**); 1 mm (**b**).

**Figure 3.**
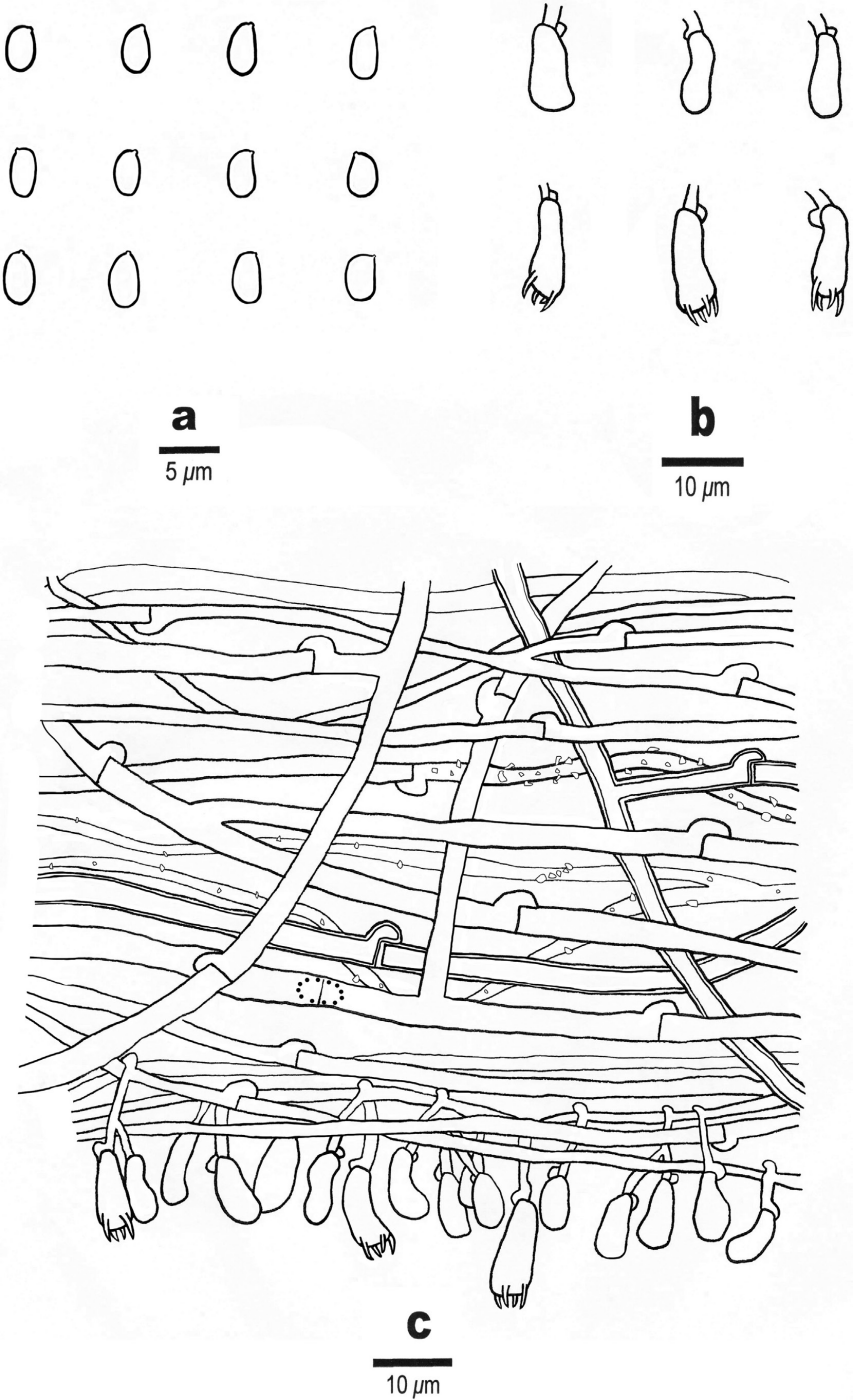
Microscopic structures of *Leptosporomyces caeruleogriseum* (holotype, BJFC 060084) **a**. Basidiospores; **b**. Basidia and basidioles; **c**. a section of basidioma.

##### Etymology.

*Caeruleogriseum* (Lat.): refers to the species having bluish gray hymenophore when fresh.

##### Description.

***Basidiomata***. Annual, resupinate, coriaceous, without odor or taste when fresh, becoming soft membranous and lighter in weight upon drying, up to 4 cm long, 2.5 cm wide and 0.4 mm thick at the center when dry. Hymenophore slightly scabrous, hypochnoid to arachnoid. Surface bluish gray (20C4) when fresh, becoming cream (4A2) to curry yellow (4B8) upon drying; sterile margin indistinct, somewhat fibrillose, white to cream (4A2).

***Hyphal structure***. Hyphal system monomitic; generative hyphae bearing clamp connections, hyaline, IKI−, weakly CB+; tissues unchanged in KOH.

***Subiculum and Hymenium***. Subicular generative hyphae clamped, 2–3 µm in diam, thin- to slightly thick-walled, usually branched, crystals occasionally present among the hyphae, interwoven. Subhymenial hyphae 2–2.5 µm in diam, thin–walled, frequently branched. Cystidia and cystidioles absent. Basidia cylindrical–subclavate, 8.5–12 × 3.4–4.5 µm, 4-sterigmate, with basal clamp connection; basidioles abundant and dominant, in shape similar to basidia, but slightly smaller.

***Basidiospores***. Oblong ellipsoid, hyaline, thin-walled, smooth, IKI−, faintly CB+, 3–4 (–4.5) × 1.5–2 (–2.2) µm, L = 3.52 μm, W = 1.76 μm, Q = 2–2.1 (n=60/2).

***Type of rot***. White rot.

##### Additional specimen examined.

China • Guizhou, Weining County, Forests of Xiaohai Town, 26.9746°N, 104.1788°E, elevation 2200 m, on fallen branch of *Pinus yunnanensis*, 25 June 2024, Dai 28277 (BJFC048537).

##### Notes.

*Leptosporomyces caeruleogriseum* is characterised by coriaceous basidiomata with a bluish gray hymenophore surface when fresh, soft membranous and cream to curry-yellow when dry, indistinct sterile margin, encrusted generative hyphae, the absence of cystidia, oblong ellipsoid basidiospores measuring 3–4 × 1.5–2 µm.

In the phylogenetic analyses, *Leptosporomyces caeruleogriseum* is closely related to *L. galzinii*, *Byssocorticium atrovirens*, *B. caeruleum*, *B. pulchrum*, and *B. yunnanense*. The four species of *Byssocorticium* can be readily distinguished from *Leptosporomyces caeruleogriseum* by their larger basidia and differently shaped basidiospores. *Byssocorticium atrovirens* possesses basidia measuring 15–25 × 4–5 µm and subglobose basidiospores (Bernicchia et al. 2010); *B. caeruleum* has basidia 22–30 × 6–7 µm and globose, subglobose, to pyriform basidiospores (Kotiranta et al. 2012); *B. pulchrum* is characterized by basidia 25–30 × 6–7 µm and globose basidiospores (Eriksson and Ryvarden 1973); and *B. yunnanense* has basidia 16.5–32 × 5–7.5 µm and globose to ellipsoid basidiospores (Wijesinghe et al. 2025). In contrast, *L. caeruleogriseum* has much smaller basidia (8.5–12 × 3.4–4.5 µm) and oblong ellipsoid basidiospores.

Morphologically, *L. caeruleogriseum* is highly similar to *L. galzinii* by having resupinate and smooth basidiomata, similar hyphal structure, and similar basidiospores (Eriksson and Ryvarden 1976). However, *L. galzinii* has detachable basidiomata with a glaucous surface and has a distribution in Europe (Eriksson and Ryvarden 1976), whereas our new species has fully effused basidiomata with a bluish gray surface and occurs in Asia.

#### Pseudoathelia

Taxon classification

Fungi



Yu Q. Liu & S.H. He, J. Fungi 12(2, no. 153): 8 (2026)

3E54E1CC-9945-594B-9379-FE4FC38F70BE

##### Notes.

Three species, namely, *Pseudoathelia fabri*, *P. linzhiensis*, and *P. septentrionalis*, are accepted within this genus. *Pseudoathelia* was recently established to accommodate two species originally placed in *Leptosporomyces* (Liu et al. 2026). In the phylogenetic analyses, *Pseudoathelia* form an independent clade separated from other genera within Amylocorticiales. *Pseudoathelia* is recognized by merulioid, tuberculate to membranous hymenophore when fresh, fibrillose sterile margin and ellipsoid, oblong ellipsoid to broad cylindrical basidiospores, and growth on gymnosperm wood (Liu et al. 2026).

#### Pseudoathelia fabri

Taxon classification

Fungi



Y.J. Cui, Chao G. Wang & Y.C. Dai sp. nov.

AB028C16-E7F7-5ACF-B385-2ADFC8EFD049

863720

Figs 4, 5

##### Holotype.

China • Xizang, Rikaze, Yadong County, Forests from Pasha Falls to Nathu La Gate, 27°20.184'N, 88°57.972'E, elevation 3,200 m, on fallen branch of *Abies fabri*, 16 October 2024, Dai 31684 (BJFC 051943).

**Figure 4.**
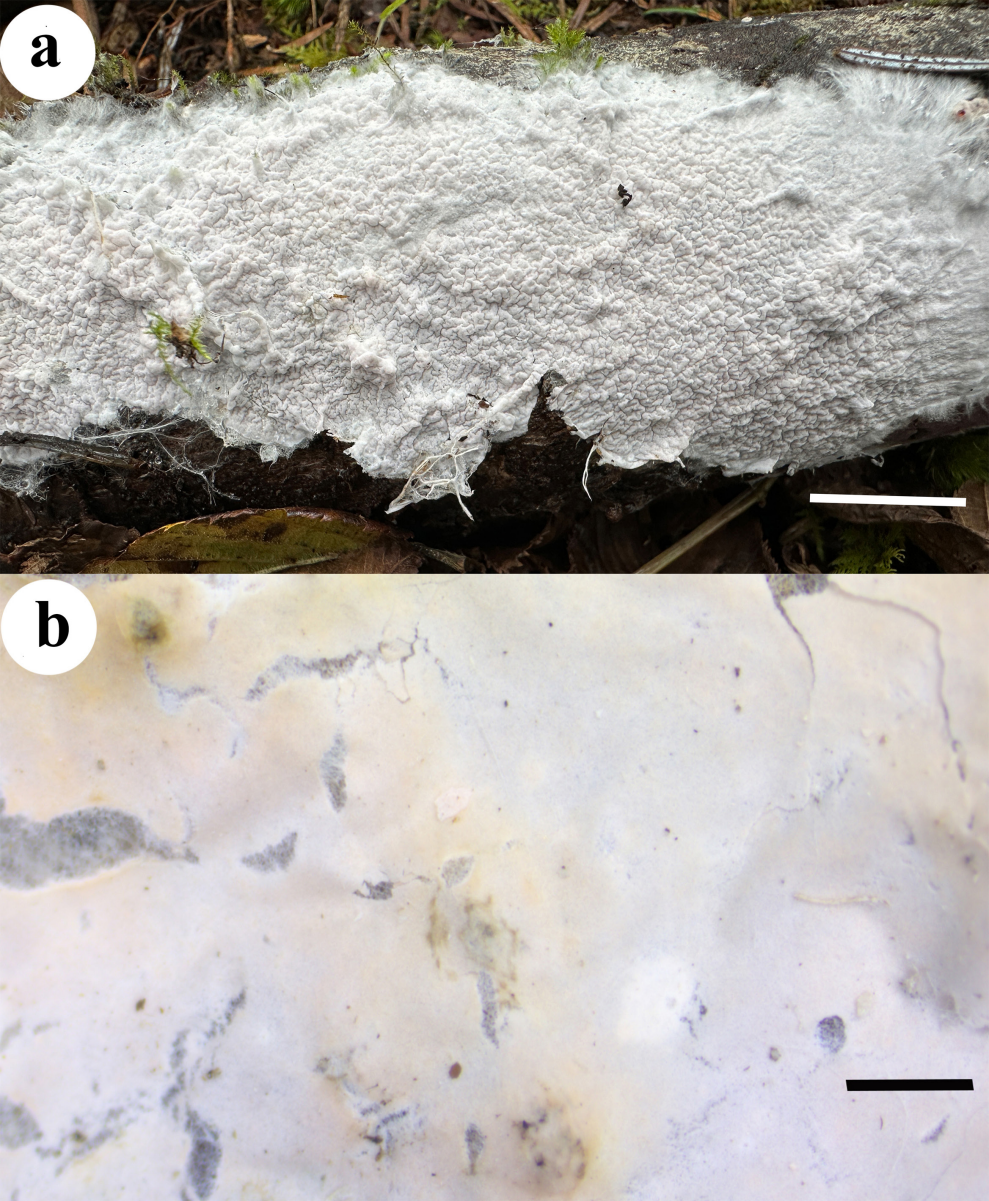
Basidiomata of *Pseudoathelia fabri* (holotype, BJFC 051943). Scale bars: 1 cm (**a**); 1 mm (**b**).

**Figure 5.**
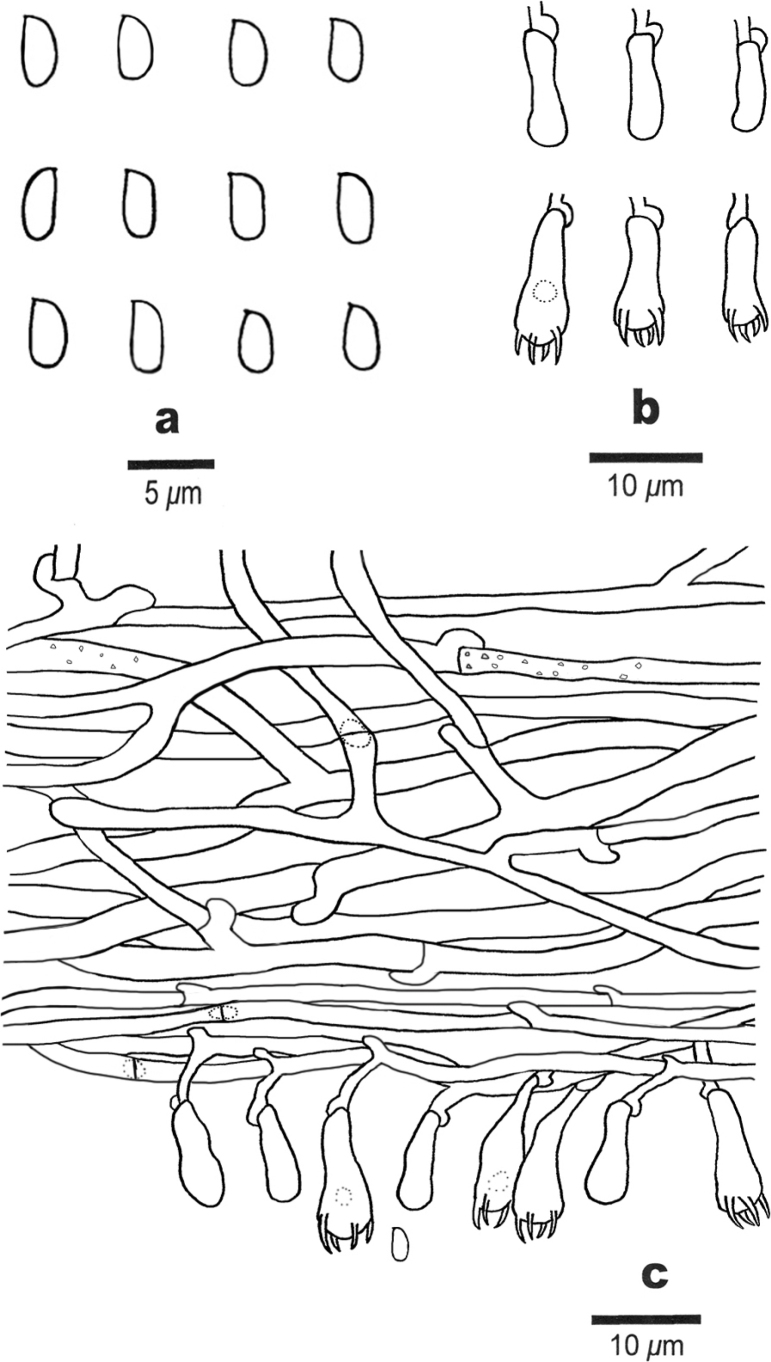
Microscopic structures of *Pseudoathelia fabri* (holotype, BJFC 051943) **a**. Basidiospores; **b**. Basidia and basidioles **c**. A section of basidioma.

##### Etymology.

*Fabri* (Lat.): refers to the species growing on *Abies fabri*.

##### Description.

***Basidiomata***. Annual, resupinate, ceraceous to leather and soft, without odor or taste when fresh, becoming brittle membranous and lighter in weight upon drying, up to 11 cm long, 3 cm wide and 0.4 mm thick at the center when dry. Hymenophore merulioid and white when fresh, becoming cream (4A2) to salmon (6A4) upon drying; sterile margin fimbriate, concolorous to hymenophore.

***Hyphal structure***. Hyphal system monomitic; generative hyphae bearing clamp connections, hyaline, IKI−, CB−; tissues unchanged in KOH.

***Subiculum and Hymenium***. Subicular generative hyphae clamped, 1.8–5 µm in diam, thin walled, branched, interwoven, occasionally encrusted with small granules. Subhymenial hyphae 1.8–3 µm in diam, thin-walled, frequently branched. Cystidia and cystidioles absent. Basidia clavate, 16.5–19.5 × 4.7–6.5 µm, 4-sterigmate, with basal clamp connection, with or without oily contents; basidioles of similar shape to basidia but smaller.

***Basidiospores***. Oblong ellipsoid, thin-walled, smooth, IKI−, CB−, (4.0–)4.1–5.5(–5.8) × (1.5–)1.8–2.4(–2.6) µm, L = 4.77 μm, W = 2.05 μm, Q = 2.29–2.32 (n=60/2).

***Type of rot***. White rot.

##### Additional specimen examined.

China • Xizang, Rikaze, Yadong County, Forests from Pasha Falls to Nathu La Gate, 27.3374°N, 88.9785°E, elevation 3100 m, on fallen branch of *Abies fabri*, 16 October 2024, Dai 31693 (BJFC 051952).

##### Notes.

Phylogenetically, *Pseudoathelia fabri* is related to *Pseudoathelia septentrionalis* and *Pseudoathelia linzhiensis*. However, *P. septentrionalis* is readily distinguished from *P. fabri* by its longer basidiospores (6–6.5 × 1.5–2 µm vs. 4.1–5.5 × 1.8–2.4 µm, Bernicchia et al. 2010) and smaller basidia (12–15 × 3–4 µm vs. 16.5–19.5 × 4.7–6.5 µm, Bernicchia et al. 2010). *Pseudoathelia linzhiensis* differs from *P. fabri* by its smaller basidia (11.5–13.5 × 3.2–3.8 µm vs. 16.5–19.5 × 4.7–6.5 µm, Zhou et al. 2024).

*Pseudoathelia fabri* is morphologically similar to *P. septentrionalis* by the merulioid hymenophore which becomes smooth and more or less cracked when dry as well as a fibrillose sterile margin with white rhizomorphs, but the latter species has subcylindrical to subfusiform basidiospores (5–6.5 × 1.5–2 µm vs. 4.1–5.5 × 1.8–2.4 µm, Eriksson and Ryvarden 1975). In addition, although *P. fabri* and *P. linzhiensis* share a similar distribution in southwestern China, the latter has smooth hymenophore when fresh and smaller basidiospores (3.8–4.3 × 1.7–2 µm vs. 4.1–5.5 × 1.8–2.4 µm, Zhou et al. 2024).

## Discussion

In recent years, numerous taxa of wood-inhabiting fungi have been described and recorded worldwide (Hyde et al. 2024; Thiyagaraja et al. 2025; Wijayawardene et al. 2025). Substantial progress in the taxonomy and diversity of wood-inhabiting fungi has also been achieved in China (Jia et al. 2014; Wang et al. 2023; Zhang et al. 2023; Dong et al. 2024; Liu et al. 2025; Wang et al. 2025; Yang et al. 2025; Zhao et al. 2025a, 2025b; Cao and Zhang 2026).

In the present study, phylogenetic analyses based on the combined ITS+nLSU dataset revealed the relationships among genera of Amylocorticiales and Atheliales within Basidiomycota (Fig. 1). Fourteen genera, including *Pseudoathelia*, are currently accepted in Amylocorticiaceae within Amylocorticiales. *Pseudoathelia*, typified by *P. septentrionalis*, is characterized by athelioid basidiomata, a smooth white to yellowish hymenophore, a fimbriate margin, the presence of rhizomorphs, a monomitic hyphal system with clamped generative hyphae, and oblong-ellipsoid, subcylindrical to ellipsoid basidiospores (Liu et al. 2026; this study). Prior to the present study, two species, *P. linzhiensis* and *P. septentrionalis*, had been recognized in *Pseudoathelia* (Liu et al. 2026). In the present study, two specimens collected from southwestern China (Dai 34684 and Dai 31693), formed a well-supported lineage distinct from previously known species of *Pseudoathelia* and are therefore proposed as a new species *P. fabri*. The new species is characterized by merulioid hymenophore with a white surface when fresh, fimbriate sterile margin, and oblong ellipsoid basidiospores measuring 4.1–5.5 × 1.8–2.4 µm.

Two specimens, Dai 28277 and Dai 38825, collected from Xizang and Guizhou in southwestern China, are characterized by coriaceous bluish gray fresh basidiomata with a smooth hymenophore, indistinct sterile margin, encrusted generative hyphae, and oblong ellipsoid basidiospores measuring 3–4 × 1.5–2 µm. These characteristics are generally consistent with the definitions of *Leptosporomyces galzinii*, *L. mundus* (H.S. Jacks. & Dearden) Jülich, *L. fuscostratus*, and *L. raunkiaeri*, all of which possess whitish to bluish gray basidiomata and narrowly ellipsoid to subcylindrical basidiospores (Jackson and Dearden 1951; Eriksson and Ryvarden 1976; Hjortstam and Ryvarden 1985; Kotiranta and Saarenoksa 1990). However, the two specimens formed a distinct lineage separate from these four species in the ITS phylogeny. Although nLSU sequence data are currently unavailable for *L. caeruleogriseum*, the combination of ITS sequence data and morphological evidence supports its recognition as a distinct species.

Notably, species of *Leptosporomyces* and *Pseudoathelia* appear to show a preference for coniferous substrates. For instance, *L. caeruleogriseum* was collected on fallen branches of *Pinus yunnanensis*; *L. galzinii* occurs mainly on decaying coniferous wood (Sulistyo et al. 2021); *L. fuscostratus* has been recorded on *Picea* (Bernicchia et al. 2010); and *L. mundus* in northern Europe is predominantly associated with dead pine trunks in old-growth coniferous forests (Kunttu et al. 2016). Similarly, species of *Pseudoathelia* are wood-inhabiting fungi causing white rot on coniferous substrates, *P. fabri* grows on fallen branches of *Abies fabri*, *P. linzhiensis* on fallen trunks of *Abies* (Zhou et al. 2024), and *P. septentrionalis* on *Picea* (Bernicchia et al. 2010). These observations suggest that coniferous woody substrates may represent an important ecological niche for species of both genera.

### Key to the species of *Pseudoathelia*

**Table:** 

1	Hymenophore smooth when fresh	***P. linzhiensis***
–	Hymenophore merulioid when fresh	**2**
2	Basidiospores subcylindrical to fusiform, 6–6.5 × 1.5–2 µm	***P. septentrionalis***
–	Basidiospores oblong ellipsoid, 4.1–5.5 × 1.8–2.4 µm	***P. fabri***

### Key to the species of *Leptosporomyces*

**Table:** 

1	Basidiospores subglobose or globose	**2**
–	Basidiospores ellipsoid, cylindric or suballantoid	**3**
2	Basidiospores subglobose, 2.5–3.5 µm in diam	***L. juniperinus***
–	Basidiospores globose, 2.4–3.1 µm in diam	***L. globosus***
3	Cystidia abundant, cylindrical, 25–35 × 3–3.5 µm	***L. mundus***
–	Cystidia absent	**4**
4	Sterile margin with rhizomorphs	**5**
–	Sterile margin without rhizomorphs	**7**
5	Basidiospores < 2 μm in width	***L. fuscostratus***
–	Basidiospores > 2 μm in width	**6**
6	Hymenophore pale pink	***L. roseus***
–	Hymenophore cream to light ochraceous	***L. luteofibrillosus***
7	Basidia > 15 μm in length	**8**
–	Basidia < 15 μm in length	**10**
8	Hymenophore brown when fresh	***L. adnatus***
–	Hymenophore whitish, cream or yellowish when fresh	**9**
9	Basidiospores ellipsoid, 4–5.5 × 2–2.5 µm	***L. crucelliger***
–	Basidiospores cylindrical, 5–6.5 × 2.3–2.6 µm	***L. montanus***
10	Basidiospores > 4 μm in length	**11**
–	Basidiospores < 4 μm in length	**12**
11	Hyphae without clamp connections	***L. muscigenus***
–	Hyphae with clamp connections	***L. raunkiaeri***
12	European species	***L. galzinii***
–	Asian species	**13**
13	Hymenophore bluish gray	***L. caeruleogriseum***
–	Hymenophore whitish	***L. thindii***

## Supplementary Material

XML Treatment for Leptosporomyces

XML Treatment for Leptosporomyces caeruleogriseum

XML Treatment for Pseudoathelia

XML Treatment for Pseudoathelia fabri

## Additional information

### Artificial Intelligence (AI) use

The authors accept full responsibility for the content of the manuscript, including the disclosure of any use of AI.

No AI tools were used in the preparation of this manuscript.

### Author ORCIDs

Y.-J. Cui https://orcid.org/0009-0000-8706-6771

C.-G. Wang https://orcid.org/0000-0003-4381-5720

Y.-C. Dai https://orcid.org/0000-0002-6523-0320

Y.-D. Wu https://orcid.org/0000-0003-1295-4015

### Data availability

All of the data that support the findings of this study are available in the main text or Supplementary Information.

## Supplementary materials

Supplementary material 1 A list of species, locality, specimens, and GenBank accession numbers of sequences used in this study Data type docx https://binary.pensoft.net/file/1684640This dataset is made available under the Open Database License (http://opendatacommons.org/licenses/odbl/1.0/). The Open Database License (ODbL) is a license agreement intended to allow users to freely share, modify, and use this Dataset while maintaining this same freedom for others, provided that the original source and author(s) are credited. Yu-Jin Cui, Chao-Ge Wang, Yu-Cheng Dai, Ying-Da Wu
