# Supplementary material for: Two new corticioid species in Amylocorticiales and Atheliales (Agaricomycetes, Basidiomycota) from Southwestern China
Source: MycoKeys. 2026 Jun 18;134:225–44. doi: 10.3897/mycokeys.134.200816 (PMC13306206; doi:10.3897/mycokeys.134.200816)
Supplement: Supplementary material 1 — A list of species, locality, specimens, and GenBank accession numbers of sequences used in this study [file mycokeys-134-225-s001.docx]

| **Table 1.** A list of species, locality, specimens, and GenBank accession numbers of sequences used in this study | | | | | | |
| --- | --- | --- | --- | --- | --- | --- |
| Species name | Locality | Sample/Voucher | GenBank Accession no. | | Reference |  |
|  |  |  | ITS | nLSU |  |  |
| *Agroathelia rolfsii* | Argentina | ATCC 201126 | AF499018 | AF499019 | Genbank | |
| *Amphinema byssoides* | Sweden | MRyberg | GQ162810 | — | Kotiranta et al. (2012) | |
| *A. byssoides* | Sweden | MR 00333 | LR694190 | LR694167 | Sulistyo et al. 2021 | |
| *A. diadema* | Norway | JS 25999 | GQ162811 | — | Kotiranta et al. (2012) | |
| *Amyloathelia crassiuscula* | Sweden | GB-K169-796 | DQ144610 | DQ144610 | Genbank | |
| *Amyloceraceomyces angustisporus* | China | He 2844* | MK520873 | MK491338 | Bernicchia and Gorjón (2010) | |
| *A. angustisporus* | China | He 2824 | MK520872 | MK491337 | Bernicchia and Gorjón (2010) | |
| *Amylocorticiellum subillaqueatum* | UK | KM 165142 | MZ159402 | — | Genbank | |
| *A. molle* | Antarctica | BAFCcult 4706 | MW370510 | MW368666 | Gaiser et al. (2021) | |
| *Amylocorticium ellipsosporum* | China | He 4457* | MK520876 | MK491341 | Genbank | |
| *A. indicum* | China | He 9605 | PX654313 | — | Genbank | |
| *A. subsulphureum* | Sweden | GB-M-Ryberg | DQ144611 | DQ144611 | Genbank | |
| *A. subsulphureum* | USA | CFMR-HHB 13817 | GU187506 | GU187562 | Binder et al. (2010) | |
| *Amylophanerochaete hainanensis* | China | He 7950* | PV185713 | PV211328 | Liu et al. (2026) | |
| *Amyloxenasma allantosporum* | France | SREF 166 | MN660447 | — | Maunoury et al. (2020) | |
| *Anomoloma albolutescens* | USA | CFMR-L 6088 | GU187507 | GU187563 | Binder et al. (2010) | |
| *A. albolutescens* | China | Wei 2772 | KT954951 | — | Song et al. (2016) | |
| *A. luteoalbum* | China | Cui 2687* | KT954961 | KT954975 | Song et al. (2016) | |
| *A. rhizosum* | China | Cui 10618 | KT954960 | KT954974 | Song et al. (2016) | |
| *A. submyceliosum* | China | Cui 2942* | KT954965 | — | Song et al. (2016) | |
| *Anomoporia bombycina* | USA | CFMR-L 6240 | GU187508 | GU187564 | Binder et al. (2010) | |
| *A. bombycina* | USA | DK 15-109 | OL436819 | — | Genbank | |
| *A. vesiculosa* | Japan | O-M-Nunez 934 | DQ144617 | DQ144617 | Genbank | |
| *Athelia decipiens* | Sweden | F 3008 | PQ653160 | — | Genbank | |
| *A. epiphylla* | Sweden | KHL 13899 | MT305996 | MT305996 | Sulistyo et al. (2021) | |
| *A. epiphylla* | China | LWZ 20201012-13 | ON897834 | — | Liu et al. (2024) | |
| *Atheliella conifericola* | China | LWZ 20191104-37 | OR557254 | OR527281 | Liu et al. (2024) | |
| *A. conifericola* | China | Dai 39186 | **PZ395132** | **PZ395117** | **This study** | |
| *A. conifericola* | China | Dai 27095 | **PZ395133** | **PZ395118** | **This study** | |
| *Athelopsis albofarinaceus* | China | CLZhao 31353* | PV254829 | PV544344 | Wijesinghe et al. (2025) | |
| *A. baculifera* | France | CBS 203-54 | MH857293 | MH868823 | Vu et al. (2019) | |
| *A. glaucina* | Sweden | KHL 11901 | GU187495 | GU187662 | Binder et al. (2010) | |
| *A. glaucina* | Sweden | GB 0058723 | LR694196 | LR694173 | Sulistyo et al. (2021) | |
| *A. subglaucina* | China | LWZ 20180512-13* | OR557255 | OR527279 | Liu et al. (2024) | |
| *A. subinconspicua* | Sweden | GB 0058732 | LR694197 | LR694174 | Sulistyo et al. (2021) | |
| *Byssocorticium atrovirens* | Sweden | BS 1710033 | LR694198 | LR694175 | Sulistyo et al. (2021) | |
| *B. atrovirens* | Sweden | GB 0078129 | LR694199 | LR694176 | Sulistyo et al. (2021) | |
| *B. caeruleum* | Finland | RS 09400* | GQ162814 | Q162814 | Kotiranta et al. (2012) | |
| *B. pulchrum* | Sweden | GB 0078135 | LR694200 | LR694177 | Sulistyo et al. (2021) | |
| *B. yunnanense* | China | CLZhao 33592* | PV254828 | PV544345 | Wijesinghe et al. (2025) | |
| *Ceraceomyces atlanticus* | Brazil | URM 85888* | KX685875 | NG060427 | Chikowski et al. (2017) | |
| *C. tessulatus* | Norway | KHL 16429 | KU518951 | KU518951 | Genbank | |
| *C. tessulatus* | China | He 3008 | PV185714 | PV211329 | Liu et al. (2026) | |
| *Fibulomyces fusoideus* | USA | DK 15-75 | OL436842 | — | Genbank | |
| *F. fusoideus* | USA | DK 14-174 | OL436787 | — | Genbank | |
| *F. mutabilis* | German | HGB-5753-GB | GQ162817 | GQ162817 | Kotiranta et al. (2012) | |
| *F. mutabilis* | UK | HFRGEJ 2502012 | PV690284 | — | Genbank | |
| *Lactarius deceptivus* | USA | AFTOL-ID 682 | AY854089 | AY631899 | Genbank | |
| *Leptosporomyces fuscostratus* | France | RGC 121006 | OR822106 | OR822106 | Genbank | |
| *L. fuscostratus* | China | He 9324 | PX654315 | — | Genbank | |
| *L. fuscostratus* | China | He 9393 | PX654317 | — | Genbank | |
| *L. galzinii* | Sweden | GB 0107211 | LR694202 | LR694180 | Sulistyo et al. (2021) | |
| *L. galzinii* | Sweden | KHL 11079 | EU118642 | — | Genbank | |
| *L. raunkiaeri* | USA | CFMRHHB 7628 | GU187528 | GU187588 | Binder et al. (2010) | |
| 1. *mundus* | USA | UC 2023030 | KP814378 | — | Genbank | |
| 1. *mundus* | USA | UC 2023236 | KP814466 | — | Genbank | |
| ***L. caeruleogriseum*** | **China** | **Dai 28277** | **PZ395134** | — | **This study** | |
| ***L. caeruleogriseum*** | **China** | **Dai 38825*** | **PZ395135** | — | **This study** | |
| *Leptosporomyces* sp. | USA | DK 14-119 | OL436794 | — | Genbank | |
| *Leptosporomyces* sp. | USA | UC 2023066 | KP814420 | — | Genbank | |
| *Lobulicium occultum* | Sweden | KHL 13496b | MT340827 | — | Sulistyo et al. (2021) | |
| *Piloderma bicolor* | China | He 9521 | PX654318 | — | Genbank | |
| *P. fallax* | Sweden | KHL 11155 | DQ469286 | DQ469287 | Genbank | |
| *Plicatura crispa* | France | CIRM-BRFM 2672 | PV109151 | — | Genbank | |
| *P. crispa* | China | YU 0335 | PV938625 | — | Genbank | |
| *P. crispa* | USA | FP-101310-SP | DQ534576 | AY293203 | Binder and Hibbett (2006) | |
| *P. crispa* | Norway | KHL 8615 | DQ144620 | DQ144620 | Genbank | |
| *P. nivea* | Canada | CBS 482-72 | MH860536 | MH872242 | Vu et al. (2019) | |
| *P. nivea* | China | He 9568 | PX654281 | — | Genbank | |
| *Podoserpula aliweni* | Chile | SGO 170081* | MN970529 | — | Garnica et al. (2021) | |
| *P. ailaoshanensis* | China | ZJL 2015015* | NR158915 | NG060161 | Genbank | |
| *P. pusio* | Australia | MEL 2297270 | MN970540 | — | Garnica et al. (2021) | |
| ***Pseudoathelia fabri*** | **China** | **Dai 31684*** | **PZ395136** | **PZ395119** | **This study** | |
| ***P. fabri*** | **China** | **Dai 31693** | **PZ395137** | **PZ395120** | **This study** | |
| *P. linzhiensis* | China | CLZhao 31174 | PP399152 | PP862922 | Zhou et al. (2024) | |
| *P. linzhiensis* | China | CLZhao 31183* | PP399153 | PP862918 | Zhou et al. (2024) | |
| *P. linzhiensis* | China | CLZhao 31187 | PP399154 | — | Zhou et al. (2024) | |
| *P. linzhiensis* | China | CLZhao 31190 | PP399155 | — | Zhou et al. (2024) | |
| *P. septentrionalis* | USA | UC 2023047 | KP814348 | — | Rosenthal et al. (2017) | |
| *P. septentrionalis* | Sweden | GB 0090937 | LR694203 | LR69418 | Sulistyo et al. (2021) | |
| *Russula emeticicolor* | Germany | FH 12253 | KT934011 | KT933872 | Looney et al. (2016) | |
| *Serpulomyces borealis* | Canada | UC 2023227 | KP814487 | — | Rosenthal et al. (2017) | |
| *S. rhizomorphus* | China | CLZhao 31154 | PP399151 | — | Zhou et al. (2024) | |
| *S. yunnanensis* | China | CLZhao 19070* | OQ132516 | OQ147006 | Zhou et al. (2024) | |
| *Stereopsis vitellina* | Sweden | F 703241 | LR694211 | LR694189 | Sulistyo et al. (2021) | |
| *S. vitellina* | Sweden | Gilsenius | JN649374 | JN649374 | Sjökvist et al. (2012) | |
| *Tretomyces lutescens* | Sweden | KHL 2009a | GQ162820 | GQ162820 | Kotiranta et al. (2012) | |
| *Tylospora asterophora* | Germany | UE112-LW116 | AF052554 | — | Eberhardt et al. (1999) | |
| *T. asterophora* | Germany | LW 117 | AF052555 | — | Eberhardt et al. (1999) | |
| *T. fibrillosa* | Germany | LW 13 | AF052565 | — | Eberhardt et al. (1999) | |
| *T. fibrillosa* | Germany | LW 11 | AF052564 | — | Eberhardt et al. (1999) | |
| Notes: New species and newly generated sequences are in bold; type specimens are indicated with an asterisk (*). | | | | | | |
